# Perspectives on Neuronutrition in Prevention and Treatment of Neurological Disorders

**DOI:** 10.3390/nu15112505

**Published:** 2023-05-28

**Authors:** Anastasiia V. Badaeva, Alexey B. Danilov, Paul Clayton, Alexey A. Moskalev, Alexander V. Karasev, Andrey F. Tarasevich, Yulia D. Vorobyeva, Viacheslav N. Novikov

**Affiliations:** 1 Department of Personalized and Preventive Medicine, Institute of Interdisciplinary Medicine, 107113 Moscow, Russia; paulrclayton@gmail.com (P.C.); dr.a.karasev@gmail.com (A.V.K.); tarasevich1902@gmail.com (A.F.T.); 2 Department for Nervous Diseases, I.M. Sechenov First Moscow State Medical University of the Ministry of Health of the Russian Federation (Sechenov University), 119991 Moscow, Russia; juliettae@mail.ru (Y.D.V.); vazlav001@gmail.com (V.N.N.); 3 Russian Research Clinical Center of Gerontology of the Russian National Research Medical University Named after N.I. Pirogov, 129226 Moscow, Russia; amoskalev@list.ru

**Keywords:** neuronutrition, neurological disorders, neuronutrients, brain health

## Abstract

The term neuronutrition has been proposed as part of nutritional neuroscience, studying the effects of various dietary components on behavior and cognition. Other researchers underline that neuronutrition includes the use of various nutrients and diets to prevent and treat neurological disorders. The aim of this narrative review was to explore the current understanding of the term neuronutrition as the key concept for brain health, its potential molecular targets, and perspectives of its nutritional approach to the prevention and treatment of Alzheimer’s and Parkinson’s diseases, multiple sclerosis, anxiety, depressive disorders, migraine, and chronic pain. Neuronutrition can be defined as a part of neuroscience that studies the influence of various aspects of nutrition (nutrients, diet, eating behavior, food environment, etc.) on the development of nervous disorders and includes nutrition, clinical dietetics, and neurology. There is evidence that the neuronutritional approach can influence neuroepigenetic modifications, immunological regulation, metabolic control, and behavioral patterns. The main molecular targets in neuronutrition include neuroinflammation, oxidative/nitrosative stress and mitochondrial dysfunction, gut–brain axis disturbance, and neurotransmitter imbalance. To effectively apply neuronutrition for maintaining brain health, a personalized approach is needed, which includes the adaptation of the scientific findings to the genetic, biochemical, psycho-physiological, and environmental features of each individual.

## 1. Introduction

Chronic non-infectious diseases remain the leading causes of death and disability worldwide despite the extensive development of innovative pharmaceutical technologies that are generally increasing in frequency and, in many cases, decreasing in latency. Modifiable lifestyle factors play a significant role in the prevention and therapy of these disorders, among which diet and nutritional behavior occupy a special place [1]. Dietary recommendations for corrective eating behavior and nutrient status for gastroenterological and cardiovascular diseases have been developed already [2,3]. Numerous studies show that both neuronutrients and eating behavior, in general, could impact the pathogenesis of neurological disorders and also the cognitive and emotional states of the patients [4,5].

At the same time, researchers note that nutrition in neurology has always been considered narrowly in the context of managing neurological patients with malnutrition, dysphagia [6], and alcohol-related neurological disorders [7]. Additionally, micronutrient deficiencies, particularly B2 and B12 [8,9], iron [10], and copper deficiencies [11], may result in the onset of different neurological symptoms. On the other hand, an excess of micronutrients, such as copper, can lead to the development of other neurological disorders, such as Wilson’s disease [12]. One more notable domain of clinical nutrition in neurology pertains to the application of a ketogenic diet for the management of refractory epilepsy [13] and Glucose transporter type 1 (GLUT1) deficiency syndrome [14].

Another important and well-discussed nutritional aspect in neurology is ischemic stroke prevention, as it has a lot of common risk factors with other cardiovascular disorders, which is not the subject of this review and can be read elsewhere [15,16].

Although research in nutrition science has demonstrated the potential for beneficial effects of selected nutrients and diets on such conditions as depression, anxiety, cognitive decline, and neurodevelopmental disorders [17,18,19], these findings often remain theoretical and have little application in clinical practice. Moreover, there is now an identified need for research to develop practical recommendations on nutrition and the use of neuronutrients in the prevention and treatment of various neurological disorders.

The aim of the narrative review was to explore the state of the art of the term neuronutrition as the key concept for brain health, potential neuronutritional molecular targets, and interventions as an interdisciplinary approach to the prevention and treatment of Alzheimer’s diseases, multiple sclerosis, anxiety and depressive disorders, migraine, and chronic pain.

## 2. Neuronutrition

Nutrition has traditionally been viewed as a supplier of elements for building and maintaining the human body and as a source of energy for the body’s vital functions. Research in psychoneuroendocrinoimmunology (PNEI) has broadened the horizons of the role of nutrition. According to the PNEI concept, nutrition is a tool with which the environment methodically shapes the metabolome and epigenome, and various nutrients and eating behaviors have a multifaceted effect on self-regulation, metabolism, immune system, and brain function [20].

**Figure 1:**
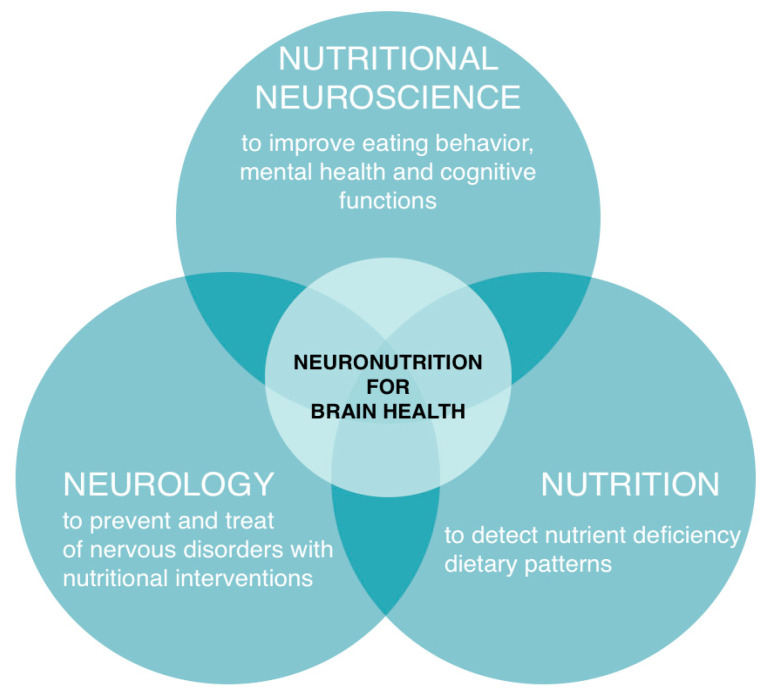
Neuronutrition definition. Neuronutrition includes a subset of nutritional neuroscience (approach to improving eating behavior [26], mental health [27], and cognitive functions [28] in healthy and sick individuals), nutrition (approach to detect nutritional status and dietary patterns in patients with neurological disorders [29]), and neurology (approach to prevent and treat neurological disorders with nutritional interventions) [30]).

A new scientific field, nutritional neuroscience, which studies the effects of dietary components, such as proteins, carbohydrates, fats, and supplements, including phytonutrients, on the central and peripheral nervous system, neurochemistry, neurobiology, behavior, and cognition, has recently emerged [21]. Some researchers suggest using the term neuronutrition as part of the nutritional neuroscience of maintaining brain health and cognitive function through dietary influence [22]. Other researchers have defined neuronutrition as not only the use of diet but also the use of various nutrients to prevent and treat disorders of the central and peripheral nervous system [23]. The first references to neuronutrition were mentioned in the context of dietary patterns influence on Alzheimer’s disease development [24]. In a broader sense, neuronutrition is an interdisciplinary area that studies the influence of various aspects of nutrition (nutrients, diet, food behavior, food environment, etc.) on brain health [25], prevention, and treatment of neurological disorders across the lifespan (Figure 1).

The future of neuronutrition, as a part of personalized and preventive medicine, is to apply neuronutritional interventions to prevent and treat brain disorders (both neurological and psychiatric), including migraine, chronic pain syndrome, epilepsy, amyotrophic lateral sclerosis, anxiety, and depressive disorders, neurodegenerative diseases (Alzheimer’s, Parkinson’s), autoimmune conditions (multiple sclerosis), and others.

Since brain dysfunction (maladaptive response to stress) contributes to the formation/progression of other chronic diseases (metabolic syndrome, arterial hypertension, irritable bowel syndrome, etc.), the neuronutritional approach may also find applications in the prevention and ancillary treatment of various somatic pathological conditions [31].

## 3. Neuronutritional Interventions

Neuronutrition includes the use of diets, functional foods (food products with specific nutritional properties), food supplements/nutraceuticals, and medications (nutrients in supradietary doses), for the prevention and treatment of neurological and psychiatric disorders [23].

**Figure 2:**
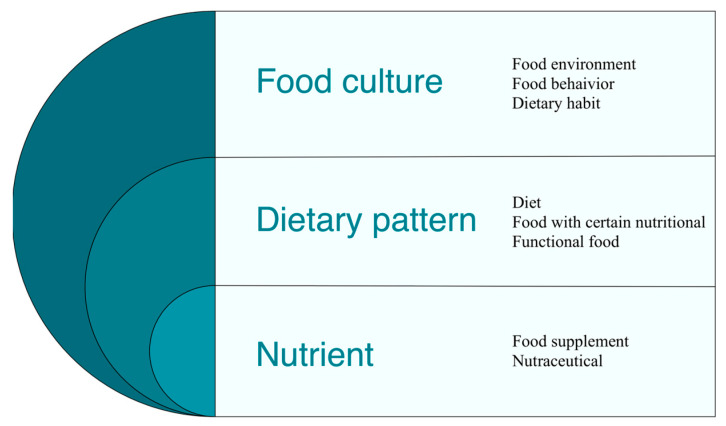
Neuronutritional interventions can be implemented through the correction of dietary culture, dietary patterns, and nutrient intake to optimize neurological health and prevent/treat neurological disorders.

As practice shows, the nervous system state, as well as whole body function, depend on the effect of individual nutrients and diets, which is largely determined by food culture. Hence, studying and forming healthy dietary habits, optimal eating behavior, and a healthy food environment are also among the areas of neuronutrition as a science (Figure 2).

### 3.1. Nutrient Interactions

Brain health preservation and neurological disorder prevention are largely associated with the suppression of signaling pathways associated with aging. Phytonutrients, such as the polyphenols apigenin, quercetin, and proanthocyanidins, have been shown to modulate and suppress many of these signaling pathways [32]. Other neuronutrients affect neuroinflammation and oxidative/nitrosative stress and/or modify neurotransmitter chemistry [33,34]

Food is a complex combination of multiple nutrients and anti-nutrients, many of which have been shown to modulate inter alia gene expression and metabolic pathways [35].

Nutraceuticals are food and/or herbal extracts utilized to ameliorate health, delay senescence, prevent diseases, and support the proper functioning of the human body [36]. This definition leads to a partial overlap with the definition of a food supplement; however, while nutraceuticals are made from food or part of a food, food supplements are single substances used alone or in mixtures with the scope of adding micronutrients when the body needs them [37].

Many food supplements and nutraceuticals have been studied in relation to nervous disease treatment and prevention. Magnesium, coenzyme Q10, feverfew, riboflavin, and phycocyanins have shown modest efficacy but a very good safety and tolerability profile in migraine treatment [38]. Diets with a low nutrient density are linked to a higher risk of cognitive decline [39]. Conversely, diets with a higher nutrient density are associated with a nutraceutical component in the Mediterranean diet and are associated with a degree of neuroprotection [40].

Food supplements were initially used to prevent and/or treat deficiencies in some essential micronutrients, thus reducing their adverse health consequences. Nowadays, this practice is more widespread, meaning that adding supplements not only covers the deficit but also helps gain a positive effect on health [41]. Many studies on food supplements’ role in the prevention and treatment of various nervous diseases are being conducted as they are safe but, at the same time, can be efficient in some areas where pharmaceutical pharmacology has been unproductive. Nicotinamide riboside supplementation, for example, was shown to augment the NAD metabolome and induced transcriptional upregulation of processes related to mitochondrial, lysosomal, and proteasomal function in blood cells and/or skeletal muscle and improve some clinical symptoms in patients with Parkinson’s disease [42]. Pro/prebiotics can be useful in Alzheimer’s disease prevention [43].

### 3.2. Dietary Pattern

A dietary pattern is defined as the amount, proportion, variety, or combination of different foods, drinks, and nutrients in the diet and the frequency of their consumption [44].

Neuronutrition’s aim is to replace maladaptive, unhealthy dietary patterns that increase chronic disease risk development with healthy dietary patterns that promote brain health [45].

According to the neuronutrition concept, the dietary pattern includes functional foods, foods with certain nutritional properties, and specialized diets that have shown effectiveness in maintaining brain health and in the prevention and treatment of neurological disorders.

The antidepressant food rating was developed to identify individual foods with the highest nutrient density for depressive disorder prevention and treatment. The highest-ranking foods were oysters, mussels, leafy greens, peppers, and cruciferous vegetables [46].

Functional foods are novel foods that have been formulated so that they contain substances or live microorganisms that have possible health-enhancing or disease-preventing values and at a concentration that is both safe and sufficiently high to achieve the intended benefit. The added ingredients may include nutrients, dietary fiber, phytochemicals, fatty acids, or probiotics [47]. In Japan, a functional product in the form of yogurt based on beta lactoline that improves memory has been developed. Taking beta-lactoline for 6 weeks improved brain blood circulation, increased concentration, and memory [48].

A growing body of evidence has been accumulated on the protective effects of the Mediterranean diet in neurodegenerative disease prevention [49,50]. Adherence to a calorie-restricted diet was found to improve the quality of life and emotional state of patients with multiple sclerosis [51].

### 3.3. Food Culture

Food culture is what we do, think, and feel around food as an individual or group within contemporary social and environmental constructs [52]. This part of neuronutrition includes aspects of dietary habits, food behavior, and food environment that affect neurological disorders prevention and treatment.

Dietary habits are habitual decisions of a person or a group of people ranging from the selection of individual foods to methods of cooking and eating [53]. Dietary habit formation involves the reward system of the brain and the nucleus accumbens and other hypothalamic nuclei, which are involved in food consumption motivation, pleasure from food intake, appetite, and satiety [54]. Unhealthy dietary habits, such as regular excessive consumption of refined carbohydrates and inadequate fiber intake, that contribute to hypothalamic dysregulation and damage [55] are risk factors for Alzheimer’s disease [56], Parkinson’s disease [57], and depression [58].

Food behavior is a complex interplay of physiological, psychological, social, and genetic factors that influence meal timing, amount of food consumed, food preferences, and food choices [59]. Regulation of hunger and satiety is controlled by hypothalamic neurons. Their signals are converted into motivated behavior to meet the homeostatic needs of a person [60]. Eating disorders contribute not only to metabolic dysregulation and obesity [61] but also to chronic pain [62] and dementia patients’ condition worsening [63]. Eating disorders, for example, have also been found in patients with migraines, and skipping meals may be an early symptom of an attack rather than a migraine trigger [64].

The food environment includes both urban and domestic environments, in which a person makes the decision about nutrition, as well as healthy and unhealthy foods available in it [65]. The environment has a great influence on food choices, which are largely determined by the context in which they are made. There is evidence that higher access to fast food restaurants near a person’s home has been associated with a higher body mass index [66]. Higher grocery shopping and lower fast food restaurant availability, as well as higher income and college education, have also been found to be independently associated with higher consumption of fresh fruits and vegetables, lower consumption of fast food and soda, and lower risk of being overweight and obese. [67].

Another part of food culture is chrononutrition, a branch of nutritional science focused on studying how nutrients or mealtimes themselves can influence the circadian rhythm system in health and disease [68]. A growing body of evidence suggests that nutrient and food consumption timing can affect circadian rhythms functioning, and circadian rhythms desynchronization can negatively affect the timing and choice of food [69]. Eating at inappropriate times can disrupt circadian rhythm organization and contribute to metabolic dysregulation and chronic disease development [70]; there is a close relationship between human personality, chrononutrition, and cardiometabolic health [71]. Data have also been published on the possibilities of chrononutrition use in medicine, with intermittent fasting improving chronic pain as an example [72].

## 4. The Molecular Targets of Neuronutrition

The mechanisms underlying the effects of nutrition on the nervous system and neurological diseases are still poorly understood. There is evidence for the effects of such aspects of nutrition as vitamin and mineral intake on the synthesis of neurotrophic factors and neurotransmitters, neuroplasticity, myelination, and microglia activity [73,74].

**Figure 3:**
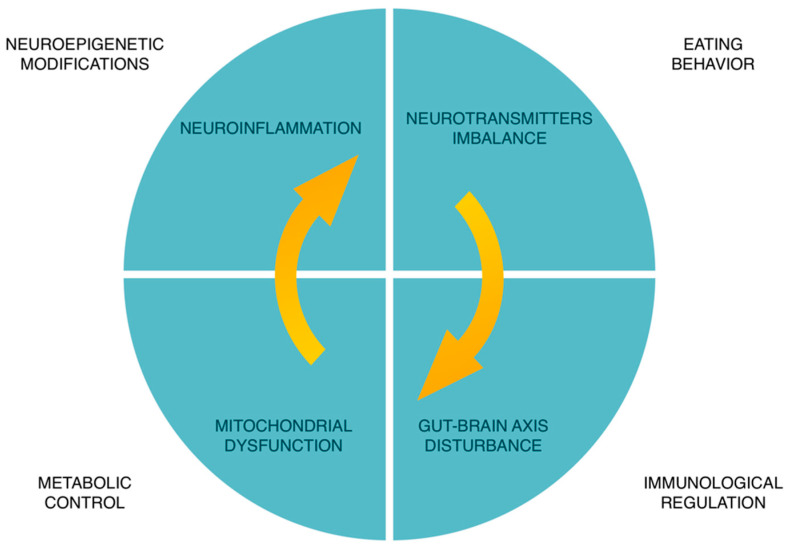
Molecular targets of neuronutrition. The main molecular targets in neuronutrition are neuroinflammation, mitochondrial dysfunction, oxidative/nitrosative stress, gut–brain axis disturbance, and neurotransmitters imbalance [75,76].

At the moment, it is assumed that neuronutritional interventions can also influence neuroepigenetics modifications, immune regulation, metabolic control, and eating behavior of patients with neurological disorders and brain health [30] (Figure 3).

There are also disease-specific neuronal targets; for example, in migraine, it is the calcitonin gene-related peptide (CGRP) and its receptors [77]; in chronic neuropathic pain—central sensitization, and in nociplastic pain—fatty acid amines [78].

### 4.1. Neuroepigenetics Modifications

Interactions between nutrition and genes are involved in brain development and function, affecting cell membranes, neurotransmitters, neurogenesis, synaptic plasticity, and metabolism in neurons [79]. Results from studies in the field of neuroepigenetics of nutrition show that diets high in sugar, trans-fats, and methionine cause changes in DNA methylation and histone modifications in brain regions, such as the hypothalamus, hippocampus, striatum, and cortex [80]. Overeating or malnutrition contributes to a chronic stressful environment and leads to neuroepigenetic reprogramming that contributes to cognitive disorders and other degenerative condition development [81]. Nutrition, being a powerful epigenetic regulator, plays an important role in preserving brain health and preventing neurological disorders through gene modification.

### 4.2. Neuroinflammation

Neuroinflammation is involved in most neurodegenerative processes [82] and pain mechanisms [83] and represents one of the common mechanisms involved in brain aging. Neuroinflammation is characterized by hyperactivation of peripheral glia, including Schwann cells, satellite glial cells in the posterior horn of the spinal cord, and trigeminal nerve ganglia, and central glia, including microglia, astrocytes, and oligodendrocytes in the spinal cord and brain [82]. A diet high in processed foods and saturated and trans fats may contribute to the promotion of low-grade inflammation and increase the risk of the development of non-communicable diseases, including neurological disorders [84].

A prospective cohort study of more than 70,000 participants shows that high consumption of ultra-processed foods was associated with a higher risk of dementia [85].

The same association was found between having depressive symptoms and a high intake of ultra-processed foods among young individuals [86]

Positive effects of nutrition on neuroinflammatory signaling pathways regulation have been found with the consumption of whole plant foods, such as berries, mushrooms, turmeric, and garlic [87]. Interactions between different components of whole foods and plant foods contribute to a synergistic effect for neuroinflammation regulation and possible prevention of neurodegeneration.

### 4.3. Immunological Regulation: Vitamin D

Vitamin D plays a crucial role in immune system regulation and can impact various neurological conditions via this mechanism. Research indicates that low levels of vitamin D are linked to cognitive decline [88], Parkinson’s disease [89], depression, Alzheimer’s disease [90], and other neurological disorders.

The connection between microbiome and vitamin D is also significant. Studies have shown that vitamin D deficiency and the microbiome can contribute to systemic and chronic inflammation, which, in turn, can increase the risk of neurological conditions development [91].

Given these findings, there is potential for vitamin D supplementation to slow down cognitive decline in Alzheimer’s disease, particularly in its early stages [92]. However, more research is needed to determine the optimal dosage of vitamin D for preventing and treating neurological disorders, as well as its mechanisms of action. Additionally, individual patient characteristics, such as age, gender, presence of some medical conditions, and other factors that may affect vitamin D levels and its impact on the body, must be taken into account.

### 4.4. Gut–Brain Axis Disturbance

Many neurological diseases, namely, Parkinson’s disease, Alzheimer’s disease, multiple sclerosis, and chronic stress, can cause changes in the bidirectional gut–brain axis, leading to abnormalities in both gut function, such as irritable bowel syndrome, and brain function [76]. In addition, dietary regimens, antibiotic intake, and bacterial and viral infections are often associated with altered gut bacterial composition and disruption of the gut–brain axis, which may contribute to the development of neurological diseases [93]. There is evidence that pro-inflammatory gut bacteria, especially Salmonella, Bacillus, Mycobacterium, E. coli, and Staphylococcus, mediated by dysbiosis, may contribute to neuroinflammation in patients with Alzheimer’s disease [94].

Gut microbiota mediators can directly regulate the excitability of primary sensory neurons of the dorsal ganglion of the spinal cord through activation or sensitization of pain-related receptors or ion channels [95]. Consumption of fruits and vegetables stimulates the production of butyrate produced by bacterial fermentation of dietary fiber in the colon, which reduces mucosal inflammation [96]. Increased permeability of the intestinal barrier is observed in the early stages of Parkinson’s disease [97]. A large database has been accumulated on the effectiveness of probiotics in patients with Parkinson’s disease for the treatment of constipation, and new studies on their positive effects on motor and cognitive disorders in such patients are appearing [98].

### 4.5. Oxidative/Nitrosative Stress and Mitochondrial Dysfunction

In addition to the negative effects of ultra-processed foods on neuroinflammation, a pro-inflammatory diet, including added sugar and saturated fats, may also contribute to oxidative stress and mitochondrial dysfunction [99].

Metabolic changes in the brain are increasingly recognized as key risk factors for the development of cognitive impairment as well as for the chronification of migraine [100]. The main aspects of these changes are energy metabolism, reactive oxygen species metabolism, and lipid metabolism [30]. Oxidative/nitrosative stress is implicated in trauma-induced brain injury, which appears to be increasingly common in contact sports [101]. The prevention and reduction in oxidative/nitrosative stress via inter alia omega-3 PUFA/amphiphilic polyphenol combinations present an intriguing and potentially valuable way forward [102].

Decreased brain energy metabolism includes mitochondrial dysfunction and systemic metabolic dysregulation, such as insulin resistance [103]. Polyphenol resveratrol can stimulate mitochondrial biogenesis and enhance autophagy, contributing to ATP production and restoration of neuronal function [104].

The development of metabolic flexibility for the prevention and therapy of neurological disorders is also promising. Several preclinical studies have already explored the potential of metabolic reprogramming of microglia in diseases, such as Parkinson’s disease, multiple sclerosis, Alzheimer’s disease, and brain aging, by affecting glucose, amino acids, or fatty acids [105]. Clinical studies on the use of nutrients, such as L-carnitine, alpha-lipoic acid, CoQ10, B vitamins, and riboflavin to correct mitochondrial dysfunction have shown their effectiveness in reducing the number and duration of attacks in migraine patients [100].

### 4.6. Neurotransmitter Imbalance

Neurotransmitter imbalance is observed in patients with Alzheimer’s disease, in which the presence of intracellular neurofibrillary tangles and senile plaques are found, including in neurons that synthesize and use acetylcholine [106]. A decrease in GABA activity has been found in anxiety disorders [107]. In depressive disorders, often associated with many neurological diseases, complex disorders of cholinergic, dopaminergic, and serotonergic transmission have been shown [108]. It is also necessary to consider that high levels of stress contribute to abnormalities in the neurotransmitter system, and as a consequence, to cognitive disorders [109]. It has been shown that nutrition can influence emotional state and cognitive functions depending on the presence of neurotransmitter precursors contained in plant and animal foods [110]. Consumption of GABA-containing tea was found to decrease stress levels in young people while increasing heart rate variability [111].

## 5. Neuronutrition and Migraine

Recently, there has been growing evidence of neuronutritional interventions to address pathogenetic mechanisms and comorbidity of migraine, a multifactorial disease that is one of the main causes of disability in the adult population worldwide [112]. On the one hand, the well-known tools of neuronutrition for migraine are the correction of eating behavior, including compliance with regular meals, weight management, adequate hydration, and elimination of common food triggers, such as alcohol, coffee, and chocolate. On the other hand, personalization in migraine nutritional management consists of the identification and correction of nutrient deficiencies and the impact of neuroinflammation and mitochondrial dysfunction [113,114].

Correction of metabolic dysregulation in patients with migraine is possible via the modern diagnostic methods of metabolomics and the targeted effect of nutraceuticals. A forward-looking interdisciplinary approach is using continuous glucose monitoring for patients with migraine to apply personal dietary patterns as the key step to developing metabolic flexibility [115].

Current evidence also suggests that the gut–brain axis influences migraine through changes in inflammatory mediators, gut microbiota profile and its metabolites, neuropeptides and serotonin pathway, stress hormones, and nutrients [116]. In addition, neuronutritional interventions have the potential to influence other links in migraine pathogenesis, including serotonergic dysfunction, CGRP levels, nitric oxide, adiponectin and leptin, hypothalamic function, and platelet aggregation [117]. In Table 1, we summarized all relevant information on the neuronutritional approach to preventive migraine management.

## 6. Neuronutrition and Alzheimer’s Disease

The pathogenesis of Alzheimer’s disease was found to be related to dietary factors; in particular, excessive saturated fat intake and vitamin E deficiency may contribute to neurodegeneration [139]. A diet low in omega-3 polyunsaturated fatty acids and antioxidants supports neuroinflammation in patients with Alzheimer’s disease and contributes to its progression [140]. In view of the lack of effective drug treatment for Alzheimer’s disease, new therapeutic targets are being actively sought, and mitochondrial dysfunction is one of the promising ones [141]. A ketogenic diet, previously used in the therapy of epilepsy, and antioxidant nutrients can affect mitochondrial dysfunction and improve the cognitive status of patients with Alzheimer’s disease [142]. Such neuronutriton interventions on the cholinergic system as vitamin B12 and folic acid supplementation, have shown effectiveness in improving the cognitive performance of patients with Alzheimer’s disease [143,144]. In Table 2, we have shown the relevant data on the neuronutritional approach to Alzheimer’s disease.

## 7. Neuronutrition and Anxiety and Depressive Disorders

Anxiety and depression are very common disorders, which not only often coexist in one patient but can be confounding factors in many other somatic disorders that can lead to a poor prognosis for the patient [158]. Nutrition plays an important role in prevention and treatment of both anxiety and depression. Diet is a modifiable risk factor for depression; thus, improving diet can reduce the burden of depressive disorders [159]. It has been found that the increase in depressive disorders in recent decades has been paralleled by a decrease in healthy lifestyles, including a deterioration in the quality of diet [160]. Nutrients, including tryptophan, vitamin B6, vitamin B12, folic acid, phenyl-alanine, tyrosine, histidine, choline, and glutamic acid, are essential for the production of neurotransmitters, such as serotonin, dopamine, and noradrenaline, which are involved in regulating neurotransmitters that determine mood, appetite, and cognitive function [161]. Marine omega-3 fatty acids regulate dopaminergic and serotonergic neurotransmission, which can reduce both depression [162] and anxiety [163]. In Table 3, there is a summary of perspective dietary patterns and nutrients that could affect the neuronutrition molecular targets involved in anxiety and depression disorders.

## 8. Conclusions

Neuronutrition is at the intersection of neuronutrtional neuroscience, nutrition, and neurology. In a broader context, it covers the impact of aspects of nutrition (food culture, dietary patterns, nutrients) on brain health at different stages of the life span. Leading molecular targets in neuronutrition are neuroinflammation, mitochondrial dysfunction, neurotransmitter imbalances, and gut–brain axis disturbance. Changing food culture, improving dietary patterns, and the use of selected neuronutrients depending on the specific neuronutritional target is a promising multidisciplinary approach to brain health, prevention, and treatment of neurological disorders. Integrating a neuronutritional approach to the management of migraine, Alzheimer’s disease, anxiety, and depressive disorders can increase the patients’ quality of life and the burden of disease, as confirmed by randomized studies.

To effectively apply neuronutrition in clinical practice, a personalized approach is needed that will cover the genetic, biochemical, psychophysiological, and environmental factors of each patient. Additionally, more studies and clinical evidence are needed to identify individual patient phenotypes, taking into account the neuronutritional targets and such neuronutritional interventions as functional foods, diets, food supplements, and nutraceuticals.

## Figures and Tables

**Table 1:** Neuronutritional interventions for preventive migraine management.

The Molecular Target of Neuronutrition	Neuronutritional Interventions	
	Dietary Patterns	Nutrients
Mitochondrial dysfunction and metabolic control	Low Glycemic Index Diet [118] Low-fat diet [119] Ketogenic diet [120] EPA ^1^ + DHA ^2^ (1.5 g/day) and reduction in omega-6 in the diet [121]	CoQ10 ^3^ (400 mg/day) [122] - CoQ10 (30 mg/day) + L-carnitine (500 mg/day) [123] Riboflavin (400 mg/day) [124] CoQ10 (150 mg/day), riboflavin (400 mg/day), magnesium (600 mg/day) [125] Omega-3 (EPA (400 mg/day) + DHA (350 mg/day)) [126]
Gut–brain axis disturbance	Elimination diet based on immunological testing (IgG+ products) [127] Gluten-free diet [128] Plant-based diet [129]	Multispecies probiotics (Bifidobacterium and Lactobacterium) [130]
Neuroepigenetics modifications	Epigenetic diet (a diet rich in methyl-donor nutrients) [131]	B6 (25 mg/day) + B9 (2 mg/day) + B12 (400 mcg/day) [132] Curcumin (1 g/day) [133]
CGRP ^4^ levels and CGRP receptor activity	MIND ^5^-diet [134]	Ginger extract (600 mg/day) [135] Magnesium citrate (600 mg/day) [136] Vitamin D (2000 IU/day) [137] Melatonin (3 mg/day) [138]

^1^ EPA—Eicosapentaenoic acid. ^2^ DHA—Docosahexaenoic acid. ^3^ CoQ10—Coenzyme Q10. ^4^ CGRP—calcitonin gene-related peptide. ^5^ MIND—Mediterranean–DASH Intervention for Neurodegenerative Delay.

**Table 2:** Neuronutritional interventions for treatment of Alzheimer’s disease.

The Molecular Target of Neuronutrition	Neuronutritional Interventions	
	Dietary Patterns	Nutrients
Neuroinflammation	Mediterranean diet [145]	Omega-3 fatty acids (2.3 g/day) [146] Correction of vitamin D status [147] Selenium (200 mcg/day) + probiotics (Lactobacillus acidophilus, Bifidobacterium bifidum, and Bifidobacterium longum) [148]
Mitochondrial dysfunction	Ketogenic diet [149] Olive oil [150]	Thiamine (400 mg/day) [151] Alpha-lipoic acid (600 mg/day) + Omega-3 fatty acids (3 g/day) [152]
Neurotransmitter imbalance	MIND ^1^ diet [153] MCT ^2^ oil (42 g/day) [154]	Ginko biloba (240 mg/day) [155] Saffron (30 mg/day) [156] Correction of magnesium deficiency [157]

^1^ MIND—Mediterranean–DASH Intervention for Neurodegenerative Delay. ^2^ MCT—Medium Chain Triglycerides.

**Table 3:** Neuronutritional interventions for management of anxiety and depressive disorders.

The Molecular Target of Neuronutrition	Neuronutritional Interventions	
	Dietary Patterns	Nutrients
Neurotransmitter i mbalance	Modified Mediterranean diet [164] Diet rich in tryptophan (10 mg/kg/day) [165]	Correction of zinc deficiency [166] Vitamin B6 (80 mg/day) [167] L-theanine (200 mg/day) [168] Magnesium (300 mg/day) + vitamin B6 (30 mg/day) [169]
Neuroinflammation	Calorie restriction [170] Mediterranean diet [171]	Omega-3 fatty acids (DHA ^2^ (720 mg/day) + EPA ^1^ (480 mg/day) [172] Correction of vitamin D deficiency [173]
Gut–brain axis disturbance	High intake of dietary fiber [174]	Probiotics (Lactobacillus reuteri NK33 and Bifidobacterium adolescentis NK98) [175] Galactooligosaccharides (7.5 g/day) [176]

^1^ EPA—Eicosapentaenoic acid. ^2^ DHA—Docosahexaenoic acid.
